# The Design and Feasibility of Optimal Treatment for Coronary Drug-Eluting Stent In-Stent Restenosis (OPEN-ISR)—A Prospective, Randomised, Multicentre Clinical Trial

**DOI:** 10.3390/jpm15020060

**Published:** 2025-02-02

**Authors:** Péter Márton Kulyassa, Balázs Tamás Németh, István Hizoh, Laura Krisztina Jankó, Zoltán Ruzsa, Zoltán Jambrik, Brúnó Bánk Balázs, Dávid Becker, Béla Merkely, István Ferenc Édes

**Affiliations:** 1 Heart and Vascular Centre, Semmelweis University, 1122 Budapest, Hungary; kulyassa.peter@semmelweis.hu (P.M.K.); nemeth.balazs.tamas@semmelweis.hu (B.T.N.); ihizoh@web.de (I.H.); balazs.bruno@stud.semmelweis.hu (B.B.B.); becker.david@semmelweis.hu (D.B.); merkely.bela@semmelweis.hu (B.M.); 2 Invasive Cardiology Division, Department of Internal Medicine, University of Szeged, 6720 Szeged, Hungary; jankol07@gmail.com (L.K.J.); ruzsa.zoltan@med.u-szeged.hu (Z.R.); jambrik.zoltan@semmelweis.hu (Z.J.)

**Keywords:** coronary intervention, coronary heart disease, ischaemic heart disease, in-stent restenosis, drug coated balloon, drug-eluting stent

## Abstract

**Introduction:** Percutaneous coronary intervention (PCI) with drug-eluting stents (DES) is a cornerstone of the management of ischemic heart disease. However, in-stent restenosis (ISR) remains a significant clinical challenge, occurring in approximately 5–10% of patients undergoing PCI. This study is designed to compare the efficacy and safety of the primary therapeutic approaches for DES-ISR, specifically drug-coated balloons (DCBs)—paclitaxel-coated balloons (PCBs) and sirolimus-coated balloons (SCBs)—with a new-generation everolimus-eluting stent (EES), contributing to the evolving field of personalized medicine. **Methods and Analysis:** This prospective, multicentre, randomised, non-inferiority trial aims to enroll 150 patients with DES-ISR, who will be randomised into one of the following: SCB, PCB, or EES. The primary endpoint comparing DCB and EES is late lumen loss (LLL) at 6 months, as measured by quantitative coronary angiography (QCA). Secondary endpoints comparing the three arms include a device-oriented composite endpoint, intraluminal gain, optical coherence tomography (OCT) measured LLL, and correlations between LLL and quantitative flow ratio (QFR). The primary endpoint will be analysed using a non-inferiority design, with a margin set at 0.25 mm, for which the sample size was calculated. Statistical analysis of the primary endpoint will be conducted on an intention-to-treat basis with a one-tailed Mann–Whitney U test with a significance level of 95. Secondary endpoints will be analysed via superiority testing using ANOVA, the Kruskal–Wallis test, logistic regression, or Fisher’s exact test, as appropriate. **Ethics and Dissemination:** The study protocol has been approved by the Medical Devices Department of the Hungarian National Institute of Pharmacy and Nutrition, ensuring compliance with ethical standards as outlined in the Declaration of Helsinki. All investigators declare no conflicts of interest related to this study. The trial is registered in ClinicalTrials.gov under the ID: NCT04862052.

## 1. Introduction

Cardiovascular disease is a leading cause of death worldwide, and ischemic heart disease represents its largest causal proportion [1]. Over the past three decades, the widespread adoption of percutaneous coronary intervention (PCI) has significantly improved patient prognosis and quality of life. PCI of native coronary disease is performed predominantly with drug-eluting stent (DES) implantation.

The current DES technologies with concomitant pharmacotherapy strive for optimal safety and efficacy, however, restenosis is still considered as the “Achilles heel” of revascularization. Based on recent data, it is responsible for 5–10 percent of PCI cases in Europe and the United States [2,3]. As it affects masses of patients, it is of paramount importance to optimise diagnostic and therapeutic modalities in post-PCI patients.

Modern stents used in PCI release antiproliferative drugs to reduce restenosis within and around the stent, primarily caused by neointimal proliferation. However, this addresses only a small proportion of causes in the realm of in-stent restenosis (ISR), as a wide range of factors, such as dimensions, mechanical, and patient characteristics, also influence its development. Thus, a personalized approach that considers patient-, stent-, and lesion-specific characteristics is mandated to further reduce the burden of DES-ISR. After addressing the potential mechanical factor contributing to restenosis, such as severe calcification beneath the stent leading to under-expansion or malposition, the final step involves the application of an additional layer of DES or the utilization of a drug-coated balloon (DCB). In the presence of flow-limiting dissection at this stage, the implantation of a new layer of DES is mandatory [4]. Conversely, if no flow-limiting dissection or untreatable mechanical factor is observed, a DCB-only strategy is viable, thereby reducing the risk of stent thrombosis and recurrent restenosis [5]. However, data comparing the DCB-only strategy to DES in DES-ISR are limited, particularly in the case of sirolimus-coated balloons (SCB). The new generation of PCB-s, which offer a more uniform drug delivery than earlier generations, similar to the recently developed SCB-s, potentially exhibits higher efficacy in cases of DES-ISR, warranting randomised investigation. The different mechanisms of action of paclitaxel and sirolimus could influence effectivity in DES-ISR, and investigations could support tailoring the choice of therapeutic options to fit each case.

Our study aims to investigate two advanced alternatives for the treatment of DES-ISR: DCB -PCB or SCB- and everolimus-eluting stent (EES) with clinically relevant outcomes to determine the optimal strategy in this scenario.

## 2. Materials and Methods

### 2.1. Design

This is a two-arm, prospective, multicentre, randomised, controlled, non-inferiority clinical trial comparing the efficacy and safety of two types of DCB, namely a PCB and a SCB, to a new layer of DES in patients with DES-ISR in whom the revascularization is performed. The trial is conducted in two large tertiary regional centres in Hungary, and enrolment is between April 2021 and December 2024.

### 2.2. Eligibility Criteria and Recruitment

Patients presenting with DES-ISR in the context of chronic coronary syndrome or acute coronary syndrome, including non-ST segment elevation MI (NSTEMI) or unstable angina (UA), are eligible for this study. Further inclusion criteria are: (1) age between 18 and 85 years, (2) signed informed consent, and (3) indicated revascularization on the DES-ISR lesion by the operator.

Exclusion criteria included (1) flow-limiting dissection, (2) edge dissection after pre-treatment, or (3) severe calcification in the target segment, resistant to plaque modification during pre-treatment, (4) stent to treat in coronary bifurcation where the covered side branch is ≥2 mm in diameter, and (5) previous target segment intervention for ISR.

Further exclusion criteria are (6) an inability or unwillingness to comply with study procedures, (7) known autoimmune disease, (8) hemodynamic instability, (9) coronary angiography performed post-sudden cardiac death, (10) pregnancy or breastfeeding, (11) planned surgical intervention within 6 months, (12) stroke within 6 months prior, (13) left ventricular ejection fraction below 30%, and (14) more than one critical lesion in a coronary angiogram.

### 2.3. Outcomes

The primary endpoint is late lumen loss (LLL) between treatment and the 6-month angiographic follow-up measured by quantitative coronary angiography (QCA) in the DES and DCB groups. Secondary endpoints include a device-oriented composite endpoint (DOCE) comprising target vessel myocardial infarction (TV MI), target vessel revascularization (TVR), and sudden cardiac death (SCD) during the follow-up period in the two groups. Additional secondary endpoints involve analyses in three separate groups of PCB, SCB, and EES containing intraluminal gain post-SCB/PCB/EES treatment, LLL at follow-up measured by optical coherence tomography (OCT) in the OCT subgroup, and delta quantitative flow ratio (QFR) between treatment and follow-up. Lesions without two analyzable angiographic projections with angles ≥25° apart or lesions without sufficient data for calibration will be excluded from QFR analysis. All the lesions available for QFR analysis will also be analysed by 2D-QCA analysis at each time point.

Additionally, if both are available, comparisons of LLL and QFR values across different times of measurement will be performed to determine correlation. Furthermore, a substudy will be conducted, to investigate how measured QFR values during screening correlate with operator decisions based on angiography images regarding the performance or deferral of revascularization. All outcomes will be evaluated by blinded investigators from both sites, and the average of their results will be used. Should a discrepancy greater than 20% be identified between the two sets of values, a third blinded assessor will conduct a review. The final value will then be calculated as the average of the three measurements.

### 2.4. Timeline

Screening is considered in patients with a history of DES implantation presenting for coronary angiography. These patients will be assessed for eligibility based on the previously detailed inclusion and exclusion criteria. If eligibility is confirmed, patients will be offered the opportunity to participate in the study during a discussion, accompanied by the provision of informational brochures. Willing participants will sign informed consent prior to coronary angiography, which will be obtained by co-investigators.

For screened patients requiring revascularization in lesions suitable for both DCB and a new layer of DES implantation, lesion preparation with the chosen device will be performed, where the appropriate opening of balloons with DES:balloon ratio of 1:1 will be attained.

Following this step, if the patient remains eligible for the study, randomization will occur. Randomization is stratified by the clinical centre to balance patient numbers using a secure online randomization system specifically developed for this study. In the DES arm, the procedure concludes with post-dilatation and angiography; in the DCB arms, it concludes with DCB treatment followed by angiography. If further intervention is deemed necessary by the operator, in cases such as novel flow-limiting coronary dissection or occurrence of other procedural complications, the patient will be withdrawn from the study.

**Figure 1:**
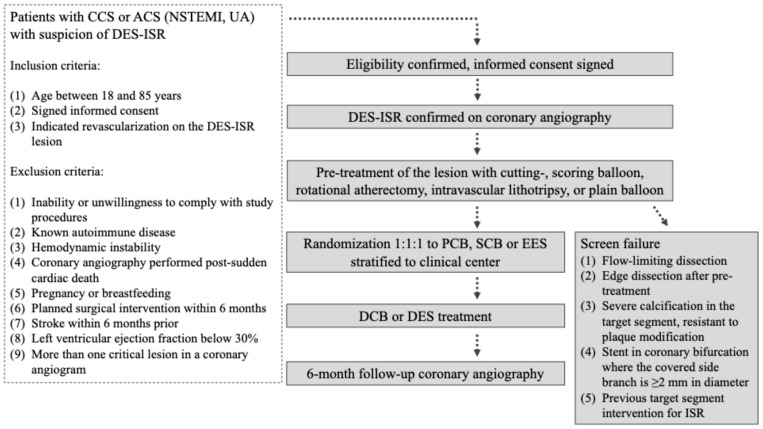
Flowchart of the OPEN-ISR trial. ACS—acute coronary syndrome, CCS—chronic coronary syndrome, DCB—drug-coated balloon, DES—drug-eluting stent, ISR—in-stent restenosis, NSTEMI—non-ST segment elevation myocardial infarction, UA—unstable angina.

Clinical and angiographic follow-up will be performed at 6 months (±30 days) post-randomization. For approximately 10–20% of patients, based on the operator’s decision that it is technically feasible, OCT imaging will be executed. It will allow a more detailed visualization of ISR plaques before the pre-treatment, and assessment of treatment results post-randomization, and during follow-up. The decision regarding OCT imaging will be made before randomization. Complications occurring during the study participation will be treated per current ESC Guidelines [6]. A summary flowchart is demonstrated in Figure 1.

### 2.5. Randomization and Assignment of Interventions

Patients will be randomised into three arms in a 1:1:1 ratio: SCB, PCB (combining paclitaxel with dextran), or EES (everolimus-eluting CrCo platform).

Randomization will be performed using a computer-generated concealed allocation sequence. An independent statistician will generate the randomization sequence, and site coordinators will implement it. Due to the nature of the interventions, blinding participants and clinicians is not feasible, however, outcome assessors and data analysts will be blinded to group assignments.

### 2.6. Sample Size Calculation and Statistical Analysis

The data on first and second-generation DES-ISR treatment are sparse, however, as similar trials were found in the literature, minor assumptions were made for sample size calculation.

In the EES arm of the RESTORE trial, an in-segment (including 5 mm proximal and distal to the stented segment) LLL of 0.15 mm (SD 0.49 mm, median FU time: 289 days) was observed [7]. Based on the data, we assumed a mean LLL of 0.15 mm in the EES arm and an SD of 0.45.

In a meta-analysis of DES-ISR treatment, PCB had an LLL of 0.25 mm (SD 0.52 mm, mean FU time: 189 days), and SCB had an in-stent LLL of 0.26 mm (SD 0.61, mean FU time 185 days) [8]. In the PCB arm of the RESTORE trial an in-segment LLL of 0.19 mm (SD 0.41, median FU time: 312 days) was published [9]. Based on the available evidence with similar results of PCB and SCB, we combined the analysis of the two devices as a DCB arm and assumed an LLL of 0.18 mm and an SD of 0.45 [9].

A patient-level meta-analysis including 2426 patients treated with first- or second-generation DES found that the optimal cut-off value of LLL for a 2-year TLR event is 0.50 mm and found an LLL over 0.50 mm to carry hazard ratio of 6.62 of incidence of TLR, regardless of target vessel segment diameter [10]. Consequently, based on the expected LLL and the LLL margin for which evidence is provided, we prespecified a 0.25 mm non-inferiority margin.

Using the above-detailed measures in a one-tailed test, with an alpha of 0.05, a power of 0.8, and a 2:1 randomization scheme, we calculated a sample size of 144: 48 for the control and 96 for the experimental arm. Considering dropout, we planned to include a total of 150 patients for the two arms.

Statistical analysis will be performed on an intention-to-treat basis. Baseline patient and disease characteristics will be summarised with detailed descriptive statistics that entail mean, median, standard deviation (SD), and interquartile range (IQR) for continuous variables, while for categorical variables, absolute and relative frequencies will be reported. Hypothesis testing of the primary endpoint will be done with a one-tailed Mann–Whitney U test with a significance level of 95. Analyses of secondary endpoints will be carried out as superiority analyses with ANOVA, Kruskal–Wallis test, logistic regression, or Fisher’s exact test as appropriate. All statistical analyses will be done in the R statistical environment. Subgroup analyses will be done based on the known predictors of restenosis, such as diabetes mellitus, reference vessel diameter, lesion length, postprocedural residual stenosis, acute gain and BMI, stent diameter, and stent length.

## 3. Discussion

While both repeated DES implantation and DCBs are employed in the treatment of DES-ISR, direct comparative data between these strategies remains limited. Multiple stent layers enhance the development of neointimal hyperplasia, which provides the substratum for developing neoatherosclerosis and presents a persistently higher risk for coronary ischemic events. On the other hand, DES implantation confers relevant mechanical advantages over the DCB-only strategy, generally resulting in larger luminal gain, while drug elution from the stent surface promotes the inhibition of neointimal hyperplasia. DCBs, by avoiding additional stent implantation, potentially mitigate the risks of a further layer of DES, albeit at the cost of higher recurrent restenosis rates. The net benefit derived from either strategy compared to the other, even under conditions that are theoretically optimal for both, remains uncertain. A more personalized approach is crucial to attain optimal outcomes. This trial aims to investigate whether DCBs can achieve comparable efficacy to repeat DES implantation while potentially reducing stent-related complications. Considering these, the selection of DCB or DES based on patient- and lesion-specific characteristics, combined with the use of adjunctive therapies such as cutting/scoring balloons, rotational atherectomy, and intravascular lithotripsy, has the potential to enhance the long-term efficacy of ISR treatment while promoting the adoption of precision medicine.

Paclitaxel is a cytotoxic drug, with a long history of utilization in DES and DCBs. It disrupts microtubule dynamics during the M phase of the cell cycle. PCBs are well established for treating ISR, with robust evidence supporting their efficacy in restenosis treatment [4,11,12].

Sirolimus, an mTOR inhibitor and cytostatic drug, inhibits DNA synthesis in the G1 phase of the cell cycle. It has demonstrated potent antiproliferative effects and has a wider therapeutic range than paclitaxel. However, until recently, the use of sirolimus in DCBs was limited due to its less lipophilic profile and larger molecular weight, which hinders its permeation to the vessel wall. Advances in encapsulating sirolimus with a lipophilic phospholipid carrier have facilitated faster uptake into the vessel wall, allowing the drug to become available during an approximately one-minute balloon inflation [8,13].

The existing literature suggests that the outcomes achieved with the two types of balloon for treating DES-ISR are similar [8]. Our approach addresses real-life situations where the operator selects whichever balloon is available. The secondary endpoints of the trial will additionally clarify the mechanisms and outcomes of different treatment strategies for DES-ISR, alongside clinical outcomes, to facilitate advanced case-by-case tool selection. Furthermore, comparing LLL achieved with the two different DCBs will offer more information regarding the comparability of these devices.

Our clinical trial was designed so that the eligible patient population would profit from both DES and DCB treatment. Conducting a clinical trial that directly compares PCBs, SCBs, and repeat DES implantation when all three options are deemed appropriate could provide valuable insights into the most effective treatment for DES-ISR in such a frequent scenario.

### 3.1. Late Lumen Loss as a Primary Endpoint

LLL measures the absolute amount of renarrowing due to vascular contraction, neointimal hyperplasia, or neoatherosclerosis. In the case of DES-ISR, LLL has been established in clinical trials to be a useful surrogate marker of harder clinical outcomes, such as TLR [10,14,15]. In a recent meta-analysis of clinical trials measuring LLL and assessing clinical outcomes such as TLR, using Youden’s index, the optimal cut-off value of 0.50 mm was suggested [15]. This value influenced the choice of the non-inferiority margin, as combined with the expected LLL rate, based on the available data, non-inferiority of the strategies would result in a lowered risk of future clinical events. There is no standardization in the trial concerning the actual target vessel segment diameter, as it is not consistently addressed or subdivided in the literature.

It is also demonstrated that LLL values seem to correlate exponentially with QFR above 0.50 mm [16]. However, the available literature on comparing LLL and QFR values is scarce, thus, we include it in our analysis.

### 3.2. Limitations

As only two centres are performing the inclusion of the study, this may limit the generalizability of the results. On the other hand, it is noteworthy that both centres involved in the trial perform more than 2000 cases of PCI annually. This combination of a low number of centres and a high volume of cases is optimal for ensuring homogeneous and high-quality results. Nevertheless, this approach may still constrain the broader applicability of the findings.

Intravascular imaging, specifically OCT, is a widely accepted method for intravascular imaging [17]. However, its use is limited to a fraction of our patient population. Despite its clinical utility, there are scenarios where conducting such examinations is not feasible. Furthermore, as it is not utilized in every case, our methods may better replicate real-world scenarios. OCT has not been fully integrated into clinical practice for cases of ISR due to various factors, including device cost, which means it does not fully reflect real-life clinical scenarios, where a personalized approach should be the driving factor.

Loss index (LLL/acute gain) is a preferred outcome measure for the comparison of DES and DCB devices, as it also takes into account acute lumen gain, which influences LLL. Nonetheless, the current literature does not substantiate its application as a surrogate for more robust clinical outcomes, such as TLR. Consequently, defining a definitive cut-off value for its use in clinical trials remains impractical, and it is not employed in the current study. This limitation is considered minor, given that the study exclusively includes successfully pretreated cases with DES:balloon size ratio of 1:1. This inclusion criterion ensures a comparable acute gain across different device types, while the stent limits the dilation, thereby reducing the impact of the device type on acute gain in DES-ISR compared to native coronary lesions.

## 4. Conclusions

This trial will aim to provide valuable insights into the treatment of in-stent restenosis by comparing various contemporary strategies and evaluating relevant outcomes.

The secondary endpoints with the correlation of various diagnostic modalities focus to reveal key characteristics of in-stent restenosis, aiding in identifying hemodynamically significant lesions.

The study’s limited inclusion of only two centres may affect the generalizability of the results, though both centres handle a large volume of PCI cases, which helps ensure high-quality outcomes.

The use of the loss index (late lumen loss/acute gain) as a measure in comparing DES and DCB devices lacks sufficient support in the literature for predicting clinical outcomes in in-stent restenosis, yet the study’s strict inclusion criteria help mitigate this limitation. This research aims to evolve a personalized approach to the treatment of DES-ISR. Such conduct is essential in this complex disease and should take into account patient-, stent-, and lesion-specific characteristics when selecting and applying therapeutic tools.

## 5. Ethics and Dissemination

### 5.1. Ethical Considerations

The study will be conducted in accordance with the Declaration of Helsinki and has received approval from the relevant ethics committee, the Hungarian National Institute of Pharmacy and Nutrition under the approval code OGYÉI/13134/2021 in March 2021. Any protocol amendments will be submitted for ethics approval and communicated to all investigators. Written informed consent will be obtained from all participants. Participant confidentiality will be maintained throughout the study, with anonymised and securely stored data. All investigators declare no conflicts of interest related to this study. Access to the final dataset will be restricted to the principal investigator and the study team. Results will be disseminated through peer-reviewed journals, conference presentations, and public summaries on the clinical trial registry. Any amendments to the protocol will be communicated by the principal investigator to all relevant stakeholders.

### 5.2. Data Collection and Management

Data collection will be conducted using standardised case report forms (CRFs) and an electronic data capture system. Data management will involve storing data in a secure database with restricted access granted only to authorised personnel, and regular audits will be performed to ensure data integrity. The technical appendix, statistical code, and anonymised dataset will be made available following the publication of the results. Every data entry will be redundantly verified by two investigators of the study. If any predetermined outcome is observed in a patient before the scheduled follow-up, the event will be counted as an outcome. Patients who withdraw from the study after randomization will be contacted via phone to collect clinical outcomes. With their consent, coronary angiograms performed outside of the study will be reviewed to gather endpoints.

### 5.3. Data Monitoring

An interim analysis will be performed at 50% of inclusion, to assess if noteworthy discrepancies in observed endpoints or adverse events occur across the cohort. The process of data collection is observed by our Data Safety Management Board (DSMB). Close communication will be adhered to between the DSMB and the clinical trial steering committee, which are both independent boards, the latter responsible for the overall conduction of the study, and the early termination if mandatory.

### 5.4. Personalized Medicine Contributions

By emphasizing tailored treatment approaches based on lesion-specific and patient-specific characteristics, this research embodies the principles of personalized medicine. The study aims to elucidate whether drug-coated balloons (DCBs) can achieve outcomes comparable to repeated drug-eluting stent (DES) implantation, potentially mitigating the risks associated with additional stent layers. Moreover, the inclusion of intraluminal gain and the correlation of late lumen loss (LLL) with the quantitative flow ratio (QFR) provides mechanistic insights into treatment responses, further advancing precision in therapeutic decision-making.

Through the integration of advanced imaging modalities, such as optical coherence tomography (OCT), and the use of quantitative angiographic metrics, the trial aspires to refine in-stent restenosis (ISR) management and inform individualized revascularization strategies. These findings have the potential to enhance patient stratification for optimal interventions, thus contributing to the broader goals of precision cardiovascular medicine.

## Data Availability

The original data presented in the study are openly available in ClinicalTrials.gov at https://clinicaltrials.gov/study/NCT04862052 or by using the study ID: NCT04862052 (accessed on 1 January 2025).
